# Global heating poses a serious threat to Australia’s birds: reply to Pacheco-Fuentes *et al*.

**DOI:** 10.1093/conphys/coac011

**Published:** 2022-03-02

**Authors:** Shannon R Conradie, Stephan M Woodborne, Blair O Wolf, Anaïs Pessato, Mylene M Mariette, Andrew E McKechnie

**Affiliations:** South African Research Chair in Conservation Physiology, South African National Biodiversity Institute, 2 Cussonia Ave, Brummeria, Pretoria 0184, South Africa; DSI-NRF Centre of Excellence at the FitzPatrick Institute, Department of Zoology and Entomology, University of Pretoria, Lynnwood Rd., Pretoria 0002, South Africa; iThemba LABS, 514 Empire Rd, Johannesburg 2193, South Africa; Mammal Research Institute, University of Pretoria, Lynnwood Rd., Pretoria 0002, South Africa; UNM Biology Department, University of New Mexico, Albuquerque, NM 87131, USA; Centre for Integrative Ecology, School of Life & Environmental Sciences, Deakin University, 75 Pigdons Road, Waurn Ponds VIC 3216, Australia; Centre for Integrative Ecology, School of Life & Environmental Sciences, Deakin University, 75 Pigdons Road, Waurn Ponds VIC 3216, Australia; Estación Biológica de Doñana (EBD-CSIC), Calle Américo-Vespucio, Edificio I, 41092 Sevilla, Spain; South African Research Chair in Conservation Physiology, South African National Biodiversity Institute, 2 Cussonia Ave, Brummeria, Pretoria 0184, South Africa; DSI-NRF Centre of Excellence at the FitzPatrick Institute, Department of Zoology and Entomology, University of Pretoria, Lynnwood Rd., Pretoria 0002, South Africa

Pacheco-Fuentes *et al.* (n.d.) argue that the effects of rapid global heating on Australia’s arid-zone avifauna will be far less severe than suggested by our recent analysis (Conradie *et al.,* 2020). We hope they are correct. But the arguments these authors present to support their view that we used unrealistically low threshold air temperature (*T*_air_) values for rapid increases in the risks of lethal dehydration or hyperthermia are unconvincing and, in several cases, not supported by the studies they cite.

Before addressing the four assumptions Pacheco-Fuentes *et al.* (n.d.) criticize in their commentary, we reiterate that the threshold *T*_air_ values we used are based on empirical studies of heat tolerance and evaporative cooling in the species concerned (McKechnie *et al.,* 2016; McKechnie *et al.,* 2017; McWhorter *et al.,* 2018; Talbot *et al.,* 2017). The relatively high vulnerability of several Australian species we modelled, particularly in terms of lethal hyperthermia, stems from the Australian passerines examined to date generally having lower heat tolerance limits [i.e. maximum environmental temperature at which body temperature (*T*_b_) can be defended at sublethal levels during acute heat exposure] compared to passerines from the arid zones of southern Africa and southwestern North America (Fig. 1).

## Assumption 1. Water is unavailable to birds throughout much of Australia’s arid zone

This assumption is one we did not explicitly make. In the case of zebra finches, we noted that the low heat tolerance of this species suggests they likely continue drinking during the heat of the day, and that this prediction was supported by Cooper *et al.*'s (2019) observations of this species drinking at air temperature (*T*_air_) > 40°C at their study site at Fowlers Gap. Conditions at this site differ in several respects from those typically experienced by zebra finches, as approximately 200 nest boxes are available throughout a 1.8-km radius vegetated area surrounding a dam (Mariette and Griffith, 2012). Moreover, *ad libitum* food is provided in artificial feeders throughout the breeding season; these feeders provide ~70% of the food provisioned to offspring (Mariette *et al.,* 2011). During Cooper *et al*.'s (2019) study, the dam was dry but zebra finches had *ad libitum* access to two artificial water sources 100 and 800 m from the dam. Thus, zebra finches at this site have much more reliable access to water and food in close proximity compared to many of their conspecifics.

We noted that artificial water sources will likely prove vital for mitigating the impacts of climate change, but we do not agree with Pacheco-Fuentes *et al.* (n.d.) that availability of water sources necessarily prevents avian mortality during extreme heat (see McKechnie and Wolf, 2010). In our discussion (Conradie *et al.,* 2020), we pointed out that lethal hyperthermia, rather than dehydration, appeared to be the major cause of mortality during historical and recent heat-related mortality events in Australia, a point also noted by Davies (1982). More recent events also support the view that a lack of water availability is often not the primary driver of mortality: during southern Africa’s first documented avian mortality event associated with extreme heat in November 2020, birds died in large numbers despite many being within a few hundred metres of the shore of the 133-km^2^ Pongolapoort Dam (McKechnie *et al.*, 2021b).

Pacheco-Fuentes *et al.* (n.d.) argue that a high density of artificial water points in parts of northwestern Australia projected to be the most challenging for zebra finches will provide a significant buffer against the impacts of rising temperatures. Yet they offer no alternate explanation for atlas data revealing a virtual absence of this species in these areas during summer (despite being a summer rainfall area), nor the large declines in reporting rates over the past two decades (Conradie *et al.,* 2020, Figure 5). Indeed, we included this analysis for zebra finches to ground-truth our predictive model for this species, which is exactly what Pacheco-Fuentes *et al.* (n.d.) call for in the last sentence of their opening paragraph.

**Figure 1:**
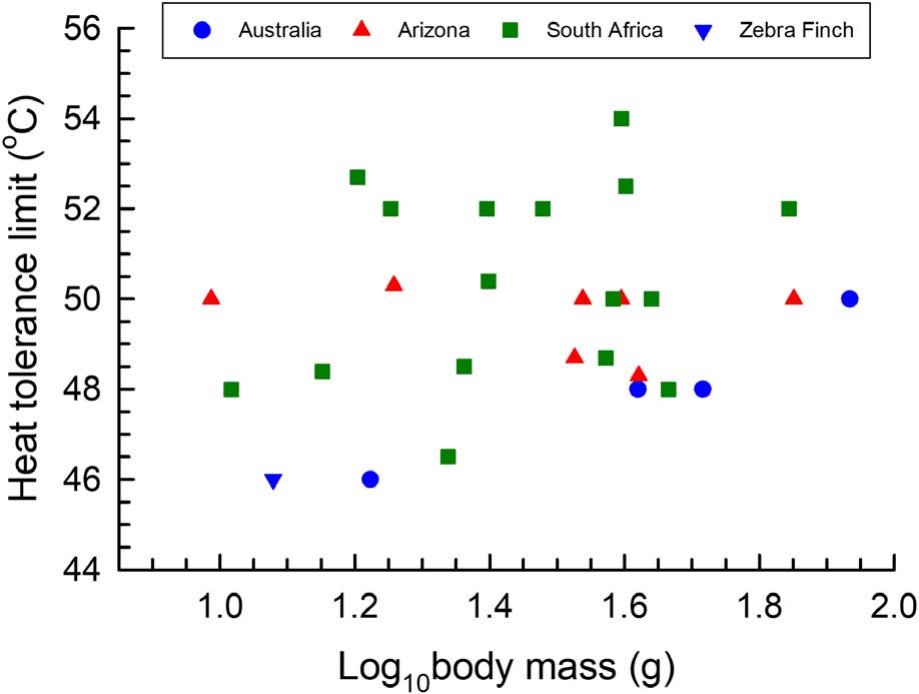
Heat tolerance limits (HTL, i.e. the maximum air temperatures tolerated under respirometry conditions) in passerines with body mass of 10–100 g occurring in arid Australia (blue circles; data from McKechnie *et al.,* 2017), the Sonoran Desert in Arizona (red triangles; data from Smith *et al.,* 2017) or South Africa (green squares; data from Czenze *et al.,* 2020; Whitfield *et al.,* 2015; and S.J. Cunningham, unpublished data). The blue downward-pointing triangle is the estimated HTL for Zebra Finches (Cade *et al.,* 1965).

## Assumption 2. Birds do not drink to replace water lost during periods of extreme heat

Pacheco-Fuentes *et al.* (n.d.) begin their critique of this assumption by implying that birds can tolerate extremely high temperatures indefinitely if sufficient water is available. Even in well-hydrated birds, however, evaporative cooling is constrained by upper limits for rates of evaporative water loss and, particularly among passerines, the metabolic costs of panting (reviewed by McKechnie *et al.*, 2021a). As already noted, the heat tolerance limits of Australian passerines investigated so far are generally low (Fig. 1).

The two studies cited by Pacheco-Fuentes *et al.* (n.d.) to support their argument that most birds of the Australian arid zone continue drinking in the middle of day even when *T*_air_ = 40–50°C actually suggest the opposite. Fisher *et al.*'s (1972) data on drinking patterns were obtained in 20 days, with only one day of maximum *T*_air_ above normothermic avian *T*_b_ (*T*_air_ = 46.5°C). For the subset of five species for which drinking data were obtained on the single day with maximum *T*_air_ = 46.5°C, three (common bronzewing, *Phaps chalcoptera*; mulga parrot, *Psephotellus varius*; Port Lincoln parrot, *Barnardius zonarius*) showed zero evidence of drinking during the heat of the day (Fisher *et al.* 1972, Figures 4–6). Spiny-cheeked honeyeaters (*Acanthagenys rufogularis*) drank mainly in the early morning, with only a handful of drinking events during the heat of the day. Data for species that drank throughout the day (including zebra finches) were typically collected on days with *T*_air_ < 36°C and none with *T*_air_ > 40°C (Fisher *et al.* 1972, Figures 4 and 6). The second study cited by Pacheco-Fuentes *et al.* (n.d.) is Davies' (1982) review of drinking behaviour in arid-zone birds. It provides no additional data for Australian species’ drinking patterns on hot days beyond those reported by Fisher *et al.* (1972). In his abstract, Davies (1982) writes: ‘*Many species have evolved hunting behaviour that enables them to remain inactive during the hottest parts of the day and thus greatly reduce the amount of metabolic heat that they need to dissipate. Flights to water are made at low ambient temperatures, either early in the morning or late in the evening*’.

## Assumption 3. Operative temperature experienced by a bird is equivalent to air temperature

Pacheco-Fuentes *et al.* (n.d.) present this assumption out of context by failing to note that we applied it only to birds resting in completely shaded microsites. Moreover, in the ‘Assumptions and limitations’ section of our discussion, we noted that *T*_e_ will indeed often differ from *T*_air_ because of factors such as partial shading (i.e. *T*_e_ > *T*_air_) or birds having access to microsites where *T*_e_ < *T*_air_ (e.g. interiors of mistletoes). Radiative heat loss to a clear sky can certainly result in *T*_e_ well below *T*_air_. But for Pacheco-Fuentes *et al.*’s argument that small birds experience daytime *T*_e_ more than 10°C below *T*_air_ to hold, the birds would need to simultaneously be completely shielded from direct and reflected solar radiation and completely exposed to the sky. These are circumstances unlikely ever encountered by birds inhabiting subtropical latitudes where the sun is approximately overhead in summer.

## Assumption 4. Physiological traits that determine thermal tolerance are fixed

In our paper, we noted that ‘*Phenotypic plasticity in physiological traits related to heat tolerance via acclimation or acclimatization has the potential to alter temperature thresholds for hyperthermia and dehydration*’. Arid-zone birds do indeed show considerable phenotypic plasticity in traits related to energy and water balance under hot conditions (e.g. Noakes and McKechnie, 2019; Noakes *et al.,* 2016; Smit *et al.,* 2013). A key issue Pacheco-Fuentes *et al.* (n.d.) overlook, however, is that for phenotypic plasticity to provide the basis for resilience to novel future environments, reaction norms for plastic traits and the range of environmental conditions over which phenotypes can be adjusted would need to extend beyond the conditions currently experienced by these species. In other words, the scope of phenotypic plasticity would need to permit birds to adjust their phenotypes to match conditions more extreme than any during their recent evolutionary history. It remains unclear whether observed avian phenotypic plasticity can be extrapolated to conditions hotter than those birds have experienced in the past. Moreover, whereas adaptive phenotypic plasticity in response to recent climate change has been demonstrated in some studies (e.g. Charmantier *et al.,* 2008), limits to reaction norms and the potential for plastic traits to be buffered from selection (e.g. Duputié *et al.,* 2015; Murren *et al.,* 2015; Oostra *et al.,* 2018) mean phenotypic plasticity is not necessarily the silver bullet suggested by Pacheco-Fuentes *et al.* (n.d.).

**Figure 2:**
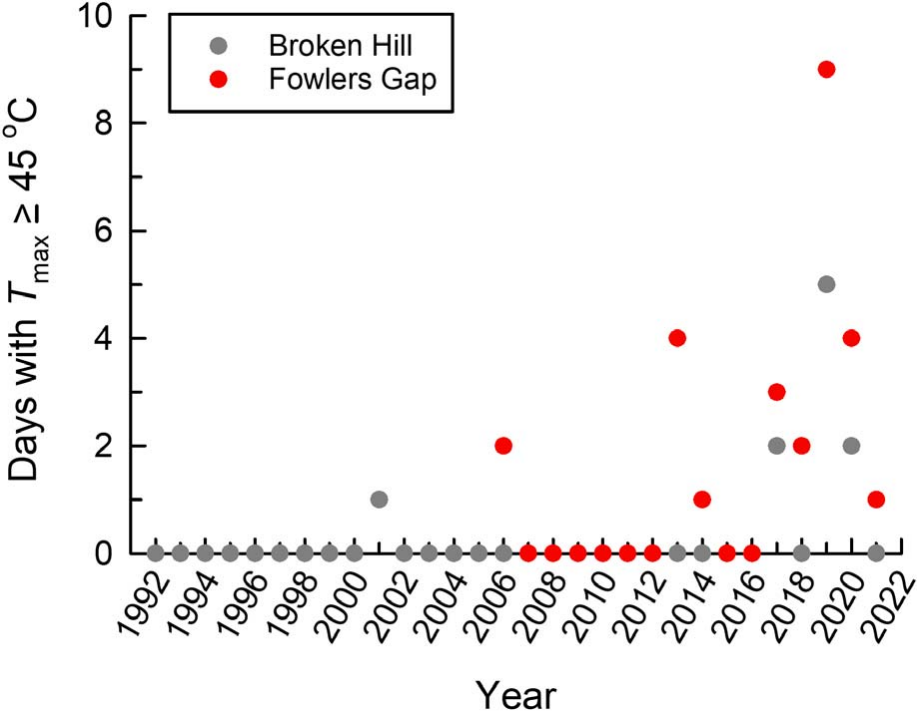
The frequency of days with maximum air temperature (*T*_max_) ≥ 45°C at Fowlers Gap (2005 to present) and Broken Hill (1991 to present) during each austral summer, taken from 1 October to 31 March. Each year indicates the midpoint of the corresponding summer; for instance, the value for 2006 is the sum of days with *T*_max_ ≥ 45°C between 1 October 2005 and 31 March 2006. Daily weather data were obtained from the Australian Bureau of Meteorology (http://www.bom.gov.au/climate/data/) for weather stations 46 128 (Fowlers Gap) and 47 048 (Broken Hill Airport).

Pacheco-Fuentes *et al.* (n.d.) cite personal observations of zebra finches persisting at *T*_air_ > 46.5°C, for example in shade under dripping stock troughs, as evidence that our estimated threshold *T*_air_ for lethal hyperthermia risk is not realistic. This argument, however, rests on an incorrect interpretation of our species-specific thresholds as precise *T*_air_ values above which no individuals of a species can survive, thereby implicitly assuming zero among-individual variation in body condition and thermoregulatory performance. To make a convincing argument for no negative effects of weather conditions we predicted as being associated with the risk of lethal hyperthermia, Pacheco-Fuentes *et al.* (n.d.) need to demonstrate that survival for the entire study population of several hundred tagged individuals (Cooper *et al.,* 2019) on extremely hot days was indistinguishable from survival on cooler days. Pacheco-Fuentes *et al.* (n.d.) also fail to make it clear that zebra finches in the Fowlers Gap population experience *T*_air_ > 45°C only rarely and mainly in the past decade (Fig. 2). The summer of 2018–2019 was the only one during which the frequency of days with maximum *T*_air_ ≥ 45°C exceeded 4 d yr^−1^ at Fowlers Gap, with the available data for Broken Hill (~120 km south) suggesting these extremely hot days were very rare prior to 2010 (Fig. 2).

In conclusion, we welcome Pacheco-Fuentes *et al.*’s commentary on our study but, for the reasons outlined above, believe their criticisms are largely unfounded. Australia has long been something of a ‘poster continent’ for avian mortality during extreme heat waves, with both historic (Finlayson, 1932; McGilp, 1932; Serventy, 1971) and recent (McCowan and Griffith, 2021; Saunders *et al.,* 2011; Sharpe *et al.,* 2021) accounts highlighting the risks of lethal effects of acute heat exposure for arid-zone birds and their embryos on very hot days. The occurrence of these events under recent and current climates lends support to our central argument that Australian arid-zone birds will face greatly increased risks of lethal hyperthermia and dehydration during extreme heat events in coming decades, unless global greenhouse gas emissions are urgently reduced.
